# Vitamin D in Inflammatory Bowel Diseases. Mechanisms of Action and Therapeutic Implications

**DOI:** 10.3390/nu14020269

**Published:** 2022-01-09

**Authors:** Filippo Vernia, Marco Valvano, Salvatore Longo, Nicola Cesaro, Angelo Viscido, Giovanni Latella

**Affiliations:** Gastroenterology Unit, Department of Life, Health and Environmental Sciences, University of L’Aquila, Piazza S. Tommasi, Coppito, 67100 L’Aquila, Italy; filippo.vernia1@gmail.com (F.V.); valvano.marco@libero.it (M.V.); salvator.longo@gmail.com (S.L.); dott.nicolacesaro@gmail.com (N.C.); angelo.viscido@univaq.it (A.V.)

**Keywords:** micronutrient, inflammation, IBD, Crohn’s disease, ulcerative colitis

## Abstract

(1) Background: Vitamin D is an immunoregulatory factor influencing intestinal homeostasis. Recent evidence supports a central role of this micronutrient in the course of Inflammatory Bowel Diseases (IBD). This narrative review aims to provide a general overview of the possible biological mechanisms of action of vitamin D and its therapeutic implications in IBD. (2) Methods: A systematic electronic search of the English literature up to October 2021 was performed using Medline and the Cochrane Library. Only papers written in English that analyzed the role of vitamin D in IBD were included. (3) Results: In vitro and animal studies reported that vitamin D signaling improves epithelial barrier integrity regulating the expression of several junctional proteins, defensins, and mucins, modulates the inflammatory response, and affects gut microbiome composition. Recent studies also suggest that vitamin D deficiency is highly prevalent among IBD patients and that low serum levels correlate with disease activity and, less clearly, with disease course. (4) Conclusions: An increasing body of evidence suggests some role of vitamin D in the pathophysiology of IBD, nonetheless the underlying mechanisms have been so far only partially elucidated. A strong correlation with disease activity has been reported but its implication in the treatment is still undefined. Thus, studies focused on this issue, the definition of vitamin D levels responsible for clinical effects, and the potential role of vitamin D as a therapeutic agent are strongly encouraged.

## 1. Introduction

Vitamin D regulates calcium and phosphate metabolism, contributing to optimal bone homeostasis [1]. Besides effects on bone, vitamin D has also been linked to a wide range of biological activities, including modulation of gut mucosal immunity and the integrity of the intestinal barrier [2,3]. Consequently, vitamin D deficiency has been associated with the activity of immune-mediated diseases, including inflammatory bowel disease (IBD). A pathogenetic role in chronic inflammatory diseases, including IBD, has also been advocated.

Vitamin D, a fat-soluble steroidal hormone, is present in humans in two main forms: vitamin D2, (ergocalciferol, from vegetables) and vitamin D3 (cholecalciferol, from animal sources). Both are provided by dietary intake and supplementation.

**Figure 1:**
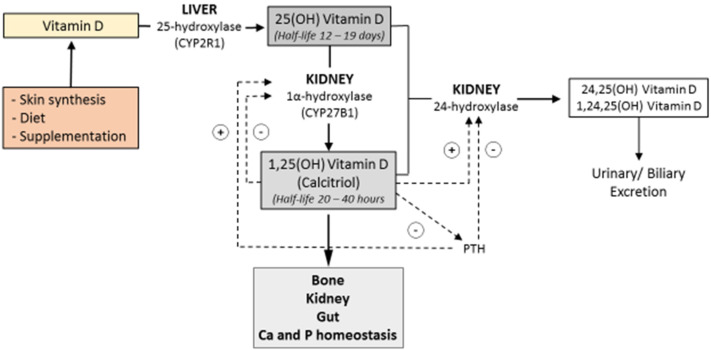
Vitamin D is synthesized in the skin in response to ultraviolet light or provided by the diet. It is first converted to 25-hydroxyvitamin D3 by hydroxylation occurring in the liver and then further converted into its active metabolite, 1,25-dihydroxyvitamin D3, in the kidney. Ca = calcium; P = phosphate; CYP2R1 = Cytochrome P450 Family 2 Subfamily R Member 1; CYP27B1= Cytochrome P450 Family 27 Subfamily B Member 1; 25(OH) vitamin D = 25-hydroxyvitamin D; 1,25 (OH) Vitamin D = 1,25-dihydroxyvitamin D.

Vitamin D3, the more biologically active compound, is synthesized in the skin in response to ultraviolet light [4]. Cholesterol is converted to 7-dehydrocholesterol in the plasma membrane of epidermal cells. The 7-dehydrocholesterol is then converted to pre-vitamin D which will be further converted into vitamin D [4]. The vitamin is then released into the circulation, bound to Vitamin D Binding Protein (VDBP) [4]. Following endogenous synthesis or intestinal absorption, vitamin D is carried to the liver where it is converted by vitamin D 25-hydroxylase into its major circulating form, 25-hydroxyvitamin D [25(OH)D]. The 25(OH)D is then further converted into its active form, 1,25-dihydroxyvitamin D [1,25(OH)_2_D], by the renal cytochrome P450 enzyme, 25-hydroxyvitamin D-1α-hydroxylase (CYP27B1) [5] (Figure 1).

Opposite effects are exerted in the kidney by 1,25-dihydroxyvitamin D3 and parathyroid hormone (PTH) on the enzymes 1α-hydroxylase and 24- hydroxylase, regulating the production and excretion of the active form of the vitamin.

Bone, kidney, and gut function, and regulation of calcium and phosphate homeostasis, result from the action of 1,25-dihydroxyvitamin D3.

Both 25(OH)D and 1,25(OH)_2_D are degraded through a third hydroxylation at carbon 24 or carbon 23 and converted by oxidation to calcitroic acid [6], which is excreted by the kidney or enters the enterohepatic circulation through bile [6].

## 2. Methods

A systematic electronic search of the English literature up to October 2021 was performed using Medline, and the Cochrane Library. The search strategy used a combination of Medical Subject Headings (MeSH) and keywords as follows: “vitamin D”, “vitamin D deficiency”, “vitamin D receptor”, “VDR”, “IBD”, “Inflammatory Bowel Disease”, “Crohn”, “ulcerative colitis”, “inflammation”, “cytokines”, “immune system”, “gut microbiota”, “intestinal barrier”, “epithelial barrier”, “epithelial permeability”, “epithelium”, “intestinal homeostasis”, “inflammatory response”, “short-chain fatty acids”, “SCFA”, “butyrate”, “response to therapy”, “therapy”, “biologics”.

Four authors selected relevant studies by screening the abstracts. Additional references were included after a review of the bibliography of the identified papers and review articles. Any difference was resolved by consensus, referring to the original articles. 

Out of 2687 citations, 119 relevant articles were selected and included in the present narrative review.

## 3. Vitamin D deficiency

Vitamin D shortage is a common health issue, but a shared definition of vitamin D deficiency is not available. Most guidelines suggest that levels below 20–30 ng/mL (50–75 nmol/L) are considered insufficient [7,8]. These figures are primarily based on the skeletal effects of vitamin D, while the minimum level required for extra-skeletal effects is less clearly defined.

According to the World Health Organization (WHO) guidelines, vitamin D shortage is further subdivided into deficiency and insufficiency, defined as serum 25(OH)D levels below 10 and 20 ng/mL, respectively [9]. However, higher cut-offs are proposed by the Endocrinological Society [8].

The prevalence of vitamin D deficiency (serum 25-OH-D < 40 nmol/L) in the general population is high, ranging between 30% [10] and 47% [11], in relation to the geographic area considered [12]. The prevalence is higher in patients at risk of vitamin D deficiency due to different causes, malabsorptive disorders, IBD included.

In a recent Italian study [13] mean vitamin D concentration in IBD patients was 18.9 ± 10.2 ng/mL. Insufficiency was present in 62% of IBD patients, and deficiency was observed in 22%. The Odds Ratio of vitamin D deficiency versus controls was 3.2, higher than that was reported in a recent meta-analysis in the IBD series (OR 1.64) [14]. The strict exclusion of subjects taking vitamin supplementation in the Italian cohort explains the difference [13].

IBD patients are known to be at particularly high risk for vitamin D deficiency [13] for several reasons including intestinal inflammation leading to impaired absorption of nutrients, bile acid malabsorption, restricted dietary intake, reduced sunlight exposure [15], or as a consequence of immunosuppressive treatment with thiopurines [16].

The prevalence of osteopenia is also high in IBD ranging from 32 to 36%, while the prevalence of osteoporosis is present in 7–15% of patients [17,18]. The mean bone mineral density (BMD) and Z-scores for IBD patients versus controls were also decreased [19]. The relative risk (RR) for bone fractures is increased in IBD patients when compared to the general population, as confirmed by two recent metanalyses [19,20]. This proves true both for the global fracture risk (RR 1.38, 95% CI 1.11–1.73) and for vertebral fractures (odds ratio 2.26, 95% CI 1.04–4.90) [19].

Low circulating levels of vitamin D is an important risk factor for osteopenia and osteoporosis in patients with IBD [21,22].

Nonetheless, the issue of vitamin D shortage in IBD is addressed by the British guidelines, but not by the more recent European Crohn’s and Colitis Organisation (ECCO) [23,24] and American College of Gastroenterology (ACG) guidelines [25,26]. Surprisingly enough the need for vitamin D supplementation was pointed out by 2017 [27], but not by 2020 [23,24] ECCO guidelines.

Besides vitamin D deficiency, reduced levels of vitamin K or magnesium, as well as increased levels of PTH secondary to low vitamin D levels, may also contribute to bone loss. Additional important risk factors for osteopenia/osteoporosis include chronic ongoing inflammation and corticosteroid therapy. It is well known that bone loss is tightly linked to immune system activation occurring during flares in chronic inflammation, mainly via TNF-α, IL-1β IL6, IL15, IL17, IFNγ, and receptor activator of nuclear factor kappa-B ligand (RANKL). Steroids may also contribute to bone loss by increasing RANKL and reducing osteoprotegerin levels. Disability or reduced physical activity caused by the active disease may be involved in the alteration of bone metabolism.

## 4. Vitamin D and Inflammation

**Figure 2:**
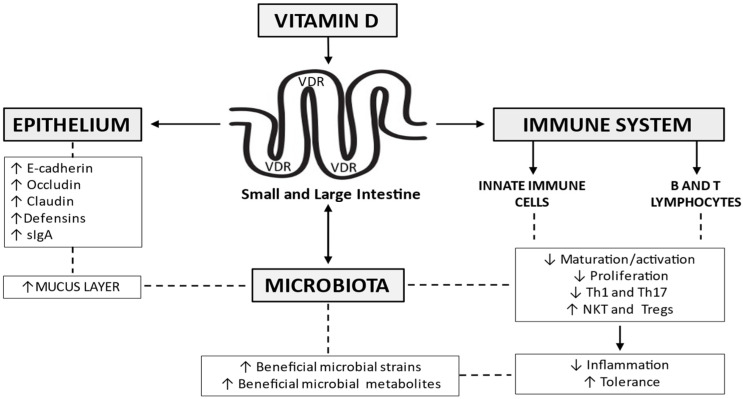
Vitamin D signaling affects the expression of several genes, regulates the immune system, and modulates the inflammatory response. It helps maintain epithelial integrity, through the regulation of tight junctions and adherens junctions’ components, as well as the release of antimicrobial peptides like the defensins. A role in the integrity of the mucus layer, as well as the composition of the gut microbiome, has been advocated. Th = T helper cells; NKT = natural killer T cells; Tregs = regulatory T cells; sIgA = secretory immunoglobulin A; VDR = vitamin D receptor.

The biological activity of 1,25(OH)_2_D is mediated by the vitamin D receptor (VDR), a member of the nuclear hormone receptor superfamily, expressed in differing organs, small and large bowel included [28]. A growing body of evidence suggests that vitamin D /VDR signaling affects the expression of several genes, regulates the immune system, and modulates the inflammatory response in experimental models of IBD [29], and humans [30,31]. Some evidence supports its role on the integrity of the mucus layer and underlying epithelium, and the composition of the microbiota (Figure 2). These issues will be addressed in the following paragraphs.

### 4.1. Intestinal Epithelial Cells and Vitamin D

VDR is highly expressed in normal gut epithelial cells [32,33]. A surface-to-crypt gradient is present, with the highest expression of VDR in the crypts [32,33]. The vitamin/VDR signaling pathway plays a primary role in the control of epithelial permeability, regulating the expression of several components of tight junctions (TJ) and adherens junctions (AJ), as well as the release of antimicrobial peptides and mucins [34] (Figure 2). In epithelial cells models, 1,25 (OH)2D3 increases the expression of E-cadherin and some TJ components, occludin, and claudins included [35]. The epithelial integrity is maintained by vitamin D/VDR signaling, also, through other mechanisms and provides a protecting effect against TNBS-induced colitis in mice [36], through inhibition of myosin light chain kinase (MLCK)-induced disruption of tight junctions. A direct role of vitamin D levels on mucus production has not been documented, and animal studies indicate that goblet cells lack VDR [37]. However, thinning of the mucus layer is present in CYP27B1−/− mice [29]. This suggests that vitamin D indirectly modulates mucus secretion, possibly favoring adequate Ca2+ assimilation [38]. The additive effects of Ca2+ and vitamin D on MUC12 expression are also involved [39]. Another hypothesis links the effects of vitamin D/VDR on microbiota and mucus production. Modulation of microbiota affects SCFA/butyrate production, and the effects of butyrate on mucus controlling genes expression are well documented [40]. Animals kept on a vitamin D-depleted diet and mouse models specifically lacking vitamin D receptor (VDR) expression in the intestinal epithelium are more susceptible to experimental colitis [32,41,42], with different mechanisms of action of the vitamin. Diet-induced vitamin D deficiency increases intestinal permeability in mice models [43], and increased disease severity was reported in a dextran sulfate sodium (DSS) animal model after the deletion of epithelial cell VDR [44]. Interestingly, VDR/IL-10 double knockout (KO) mice develop colitis after 8 weeks as compared with single IL-10- or VDR-knockout animals that remain relatively healthy at that time [45]. Conversely, the induction of epithelial VDR reduces disease activity [32]. Data however are not fully concordant, considering that gut permeability is normal in VDR deficient (VDR−/−) mice, which exhibit normal mucosal morphology both in the small [46] and in the large bowel [47]. Interestingly, in a mice model investigating the intestinal response to 1,25(OH)_2_D, the transcriptional response of VDR was reported 6 hours after a single bolus of 1,25(OH)_2_D. The effect was however limited to mature enterocytes [48]. All these data of experimental colitis in animal studies strongly support the pivotal role of vitamin D/VDR in maintaining an efficient mucosal barrier. The results on humans are less clear. Studies assessing VDR expression in IBD versus non-IBD control reported conflicting data as two studies [32,49] reported significantly lower VDR levels in inflamed IBD biopsies, while two other studies [50,51] were not able to detect overall significant differences, although VDR expression was inversely correlated with inflammatory activity. One study reported that VDR gene expression and protein immunohistochemical staining intensity were similar in different intestinal segments and between IBD patients and controls. However, a significantly lower VDR staining intensity was documented in inflamed samples versus non-inflamed epithelia, in IBD. [50]. The role of vitamin D in IBD patients, under stress conditions, was confirmed by other studies on colonic mucosa [41,52]. Mucosal inflammation was associated with a TNF-α-mediated downregulation of VDR and an up-regulation of CYP27B1 [41,52]. The paradoxical effect of vitamin D/VDR on claudin-2, as vitamin D-dependent up-regulation of claudin-2, is considered central for the paracellular absorption of Ca2+ [53]. On the other hand, vitamin D/VDR signaling, reducing claudin-2 expression, and therefore epithelial paracellular permeability, seems to be protective in IBD [49,54]. Vitamin D/VDR signaling, by inhibiting the activation of NFκB, can prevent p53 upregulated modulator of apoptosis (PUMA) induction, whose levels correlate with disease severity [55]. Moreover, in a model of DSS-induced colitis, VDR deficiency delayed mucosal healing [56]. The opposite was true for vitamin D supplementation [57,58].

### 4.2. Immune System and Vitamin D

Macrophages, dendritic cells, B cells, and T cells all express VDR and are thus vitamin D targets [59,60] (Figure 2). Furthermore, some immune cells directly produce small amounts of vitamin D. Similarly, to other extra-renal tissues, the production of 1,25D by the immune cells is modulated by the expression of Cyp27B1 [61]. Indeed, Cyp27B1 is induced via toll-like receptors or cytokines in macrophages and through T cell receptor stimulation in T cells [61,62,63,64]. Vitamin D inhibits the expression of IL-12 and toll-like receptors in dendritic cells and macrophages, as well as the dendritic cells-induced activation of T cells [65,66]. Conversely, the production of IL-10 by dendritic cells and cathelicidin in macrophages is enhanced by 1,25D [67,68]. Less innate lymphoid type 3 cells (ILC3) and lower levels of IL-22 were reported in vitamin D deficient mice compared to mice with adequate levels of vitamin D [59]. In the acquired immune system, 1,25D inhibits the proliferation of B- and T-cells [69] and inhibits the production of IL-2, interferon (IFN)-γ, IL-17, and TNF-α from T-cells [69]. The stimulation of T cells by macrophages and dendritic cells is reduced in the presence of 1,25D [66]. Conversely, 1,25D induces the production of IL-10 and other anti-inflammatory cytokines by regulatory T-cells [70] and IL-4 by Th2 cells [69] (Figure 2). Vitamin D represents an attractive target for enhancing or restoring the protective function of NKT cells [71]. Vitamin D/VDR signaling indeed contributes to the development and function of NKT cells. A lower number of NKT cells compared with wild-type (WT) mice, was observed in both VDR-deficient and 1,25D3-deficient mice (Cyp27B1−/−) [72]. Defective iNKT cell maturation in the absence of the VDR was also suggested in the same study, as KO mice fail to express NK1.1, although they express normal levels of CD122. Vitamin D treatment also leads to increased production of IL-4 and IFN-γ mice models [72]. The vitamin D-induced inhibition of T cells, reducing IFN-γ and IL-17 levels, and the induction of regulatory cells (T regs, CD8αα, and T) also supports the role of vitamin D/VDR in animal models of colitis [73]. This is in keeping with the results of experimental studies documenting that CD4 T cells from VDR KO and Cyp27B1 KO mice overproduce IFN-γ and IL-17 cells compared to wild-type CD4 cells [74]. 1,25(OH)_2_D3 suppresses the proliferation of T cells in vitro [75,76]. VDR favors FoxP3+ T reg cells, which prevent the development of experimental colitis through the production of inhibitory cytokines such as IL-10 and TGF-β [77] and FoxP3+ T reg cells are induced by 1,25(OH)_2_D3 treatments in vitro and in vivo [78]. Conversely, it has been reported that VDR KO mice have normal levels of FoxP3+ T reg cells compared to WT [79], further documenting the complexity of this issue. Other populations of regulatory T cells, such as CD8αα, are also influenced by vitamin D, thus VDR KO mice have a reduced number of CD8αα T cells in the gut, due to block in maturation and proliferation of their precursors [80]. In the clinical setting chronic activation of Th1 and Th17 cells takes place [73], possibly as IBD patients are more frequently vitamin D deficient than control subjects [14].

### 4.3. Gut Microbiota and Vitamin D

Vitamin D influences the composition of the microbiome in healthy subjects [81] and the interaction is bidirectional. Genome-wide association studies (GWAS) indicate that human VDR gene variations correlate with changes in the intestinal microbiota [82], while the absence of intestinal VDR leads to dysbiosis in mice [44]. The bacterial microbiome does not express VDR, thus VDR signaling in epithelial and immune cells mediates the effects of vitamin D on intestinal flora [83] (Figure 2). As shortage or presence of vitamin D and VDR signaling modulate several substances influencing the bacterial–host interaction, as well as innate and acquired immune response, further changes in microbiome ensue. This view is supported by a large body of evidence, but this complex mosaic of interactions needs to be better clarified. VDR knock-out mice show defective Paneth cells function [44,84], resulting in defective autophagy, granule exocytosis, and secretion of antimicrobial peptides. Thus, the lack of VDR in Paneth cells favors inflammation and susceptibility to infections in animal models [44,84]. Intestinal epithelial VDR down-regulates the expressions of ATG16L1, an IBD susceptibility gene involved in autophagy [44]. The production of lysozyme [44] and other antimicrobial peptides, such as defensin 4 [84] are reduced in VDR KO mice. Interestingly, microbiota alterations can be partially reversed by the administration of 1,25(OH)D [85]. High concentrations of vitamin D are related to increased serum cathelicidin and reduced inflammation in UC patients. Furthermore, vitamin D improves the cathelicidin antimicrobial activity in vitro against E. coli and protects against experimental colitis in vivo [86]. Conversely, the role of bacteria in modulating vitamin D levels and possible feedback interactions have been assessed by a few studies only, but it is widely accepted that commensal and pathogenic bacteria regulate VDR expression in animal models [33]. VDR signaling is indeed influenced by bacterial-produced metabolites, similar to butyrate, which is associated with increased epithelial VDR levels, in mice [44]. In turn butyrate, besides representing a primary energy substrate for colonic mucosal cells, increases the intermolecular cross-linking of fibrin chains transglutaminase- and non-transglutaminase- mediated healing processes [87]. Lithocholic acid, another intraluminal compound derived from bacterial metabolism, suppresses IL-2 production by inducing VDR signaling in T cells [88]. Interestingly, some bacterial enzymes hydroxylate steroids, and process and activate vitamin D [89]. The microbiota also influences vitamin D metabolism through fibroblast growth factor (FGF)-23 and the regulation of CYP27B1 [90]. Only a few studies evaluated the effect of vitamin D on the microbiota in UC and CD patients. Vitamin D shows a positive effect in both CD and UC patients increasing Enterobacteriaceae and reducing overall intestinal inflammation [91,92,93]. Vitamin D administration (40,000 IU, once weekly) over 8 weeks did not induce changes in alpha diversity, despite a small reduction in *Ruminococcus gnavus* in a small cohort of UC patients [91]. The increased level of *Enterobacteriaceae* was not paralleled by the significant change in *E. coli* and invasive *Fusobacterium nucleatum*. In another study, vitamin D supplementation (300,000 IU in 4 weeks) modified the gut microbiota composition in CD patients in remission, with a transient increase of beneficial bacteria such as *Alistipes*, *Roseburia*, *Parabacteroides*, and *Faecalibacterium*. No changes were noted in vitamin D deficient healthy controls. The role of vitamin D supplementation in active CD was not investigated [93]. A possible connection between the seasonal levels of serum vitamin D levels and microbiome changes was also explored [94]. Increased concentrations of *Pediococcus* spp., *Clostridium* spp., and *Escherichia/Shigella* spp., associated with the highest vitamin D levels (37.26 ng/mL) were present in summer/autumn, while *Eggerthella lenta*, *Helicobacter* spp., *Fusobacterium* spp., and *Faecalibacterium prausnitzii* were relatively less represented.

## 5. Vitamin D Supplementation and Disease Course

Hard evidence shows that mean vitamin D concentrations are lower in IBD patients than in the general population [14]. Independent predictors of vitamin D deficiency include non-Caucasian ethnicity, high BMI (>30 kg/m^2^) both in CD and UC, and IBD-related surgery in CD [95,96]. Inadequate exposure to sunlight, more so in patients with active disease, negatively affects vitamin D levels. The use of cholestyramine to treat bile acid diarrhea following distal ileum resection, also contributes to vitamin D deficiency, due to malabsorption of fat-soluble vitamins [97]. Conversely, the multivariate analysis does not show an association of steroid use with vitamin D deficiency, in IBD (*p* = 0.12 versus controls) [95]. Nonetheless, international guidelines support vitamin D supplementation in all IBD patients on steroids, to prevent the negative effect of steroids on bone metabolism [27]. A correlation between vitamin D levels and disease activity is also present [30]. Levels of 25(OH)D ≤ 25 ng/mL in a retrospective study had an AUC of 0.79–0.81 for the identification of endoscopic and histologic activity [98]. Reduced risk of postoperative endoscopic recurrence in patients with CD who underwent surgery has been reported in an observational study in patients with vitamin D > 30 ng/mL (OR 0.22, 95% CI 0.07–0.66, *p* = 0.006) [99]. Thus, vitamin D has been proposed as a potential biomarker of disease activity [98,100]. This is, at best, debatable, as vitamin D concentration reflects changes occurring over relatively long periods. Whether vitamin D supplementation represents a potential therapeutic option is controversial [100]. A well-conducted RCT including 94 CD patients, showed a non-significantly lower rate of relapse in patients treated with 1200 IU/day of vitamin D compared to the placebo group (6/46 vs. 14/48; *p* = 0.06) [92]. However, several other RCTs led to conflicting conclusions. This likely results from the small number of patients included in underpowered studies (Table 1). Moreover, the extreme variability in dosage regimens, and duration of follow-up, make results hardly comparable.

**Figure 3:**
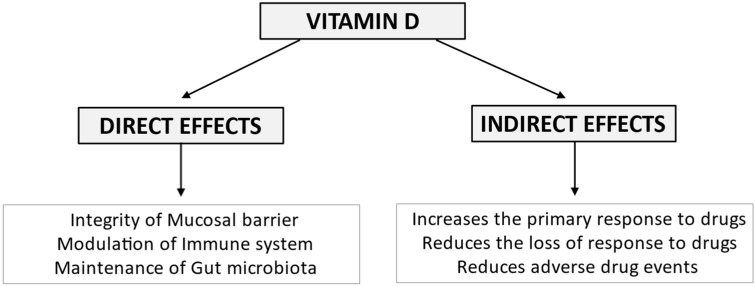
Vitamin D exerts its biological effects on the intestine in IBD maintaining mucosal barrier integrity, modulating the immune system and the composition of the gut microbiota. Emerging evidence suggests that vitamin D deficiency may unfavorably affect response to biological therapy, being associated with an increased risk of both primary non-response and secondary loss of response to the drugs. Furthermore, vitamin D deficiency may worsen corticosteroid-related osteopenia/osteoporosis and increase the risk of immunomodulator-related infections.

In a prospective study, Garg et al [114] used higher doses of vitamin D than other RCTs. In the trial, 10.000 IU of vitamin D was daily provided to 10 IBD patients. The dose was then adjusted over 12 weeks to achieve the target of 40–50 ng/mL of serum vitamin D. A significant reduction in clinical activity score was observed both in CD and UC patients [114]. Conversely, another RCT carried out in 27 CD patients in remission, treated with 2000 UI/d or placebo, showed no difference in CRP, FC, CDAI, and QoL. However, the sub-group analysis of those subjects achieving 25(OH)D concentrations of >75 nmol/L (n = 18) compared to those who did not (n = 9), documented significantly lower CRP, higher QoL, and a non-significantly lower CDAI in those with higher vitamin D levels [54]. All available RCT data were pooled in three meta-analyses [115,116,117]. Two of them reported that Vitamin D supplementation, as expected, improves the serum levels of the vitamin. It also ameliorates clinical and biochemical disease activity scores [115,116]. Conversely, the meta-analysis carried out by Guo did not confirm a decrease in disease activity indexes, despite some decrease in C-reactive protein (CRP) levels [117]. In the subgroup analysis including only observational studies by Guzman-Pardo, the Harvey Bradshaw Index improved by −1.47 points (95% CI, −2.47 to −0.47, *p* = 0.004, I2 = 0%) in CD patients, and high sensitivity CRP decreased in all sub-groups [115]. On the other hand, a sub-group analysis including four RCTs that evaluated the changes in CDAI scores following vitamin D administration did not statistically differ from controls. [115]. Therapeutic efficacy in active disease is still unclear, but vitamin D supplementation has been reported to reduce the relapse rate, irrespective of the duration of follow-up, or dosage [116]. The importance of adequate levels of vitamin D is supported by observations in IBD patients treated with biologics. Normal vitamin D levels at induction with anti-TNF-α are associated with 2.64 increased odds of remission at 3 months compared to patients with low vitamin D levels (OR 2.64, 95% CI 1.31–5.32, *p* = 0.0067) [118]. Vitamin D ≤ 25 ng/mL identified patients (6/6) losing response to biological drugs (6/50) [98]. The same holds for an increased risk of primary non-response to vedolizumab (OR 26.10, 95% CI 14.30–48.90, *p* < 0.001) and failure at 1-year follow-up (OR 6.10, 95% CI 3.06–12.17, *p* < 0.001) [119]. Overall, a role for vitamin D supplementation in the therapeutic management of IBD with direct effects on intestinal function is supported by available data (Figure 3).

Less clear are the mechanisms indirectly relating vitamin D levels, or supplementation, and the therapeutic response to biological drugs. Heterogeneity in the study design, including dosage and length of intervention, as well as the small series of patients, prevent reliable conclusions (Table 2). More RCTs with adequate size and well-defined protocols are needed.

## 6. Conclusions

Vitamin D plays an important role in maintaining intestinal homeostasis and mucosal barrier integrity, besides modulating the inflammatory immune response and the composition of gut microbiota. All these mechanisms are potentially related to the development of IBD, and some evidence suggests an influence on disease occurrence, relapse, and clinical course. Preclinical data suggest that vitamin D/VDR signaling regulates the expression of several components of tight junctions and adherens junctions, favoring the integrity of the mucosal barrier. Although the direct role of vitamin D levels on mucus production has not been documented, indirect modulation is likely. 

Vitamin D regulates immunity through direct inhibition of Th1/Th17 cells, or indirectly inducing IL-10-producing regulatory T cells. It also activates specialized cells like Paneth cells in the epithelium and promotes the expression of antimicrobial peptides. Some effects have also been described on NK cells. Vitamin D has been recently linked to changes in intestinal bacterial composition. As the microbiota does not express VDR, the effects of vitamin D on intestinal flora are supposedly mediated by epithelial and immune cells. Bidirectional effects are present, resulting from VDR functions modulated by microbial metabolites, such as butyrate. However, most studies report short-term experiments, and what happens over a long time is yet to be defined. Most data trying to elucidate the molecular mechanisms of action of vitamin D derived from studies carried out in a preclinical setting, hampering the translation of results to IBD patients. Indeed, the effects of vitamin D/VDR signaling in murine models indicate that following vitamin D administration the severity of chemically induced colitis is reduced and mucosal healing processes are more effective. Vitamin D deficiency in IBD is multifactorial, resulting from inadequate sun exposure, unnecessary dietary restrictions, and, in some instances, impaired absorption of nutrients. It is significantly more common in IBD compared to the general population with potentially relevant clinical implications. Whether vitamin D shortage results from active, long-standing disease, or represents itself a factor favoring inflammation is still to be defined. Emerging evidence, however, suggests that vitamin D deficiency may be implicated in more aggressive disease behavior and impaired response to biological therapy. If this proves true a therapeutic effect of vitamin D supplementation may be anticipated. Nonetheless, high-quality interventional RCT with an adequate baseline assessment and follow-up documenting laboratory and endoscopic improvement following vitamin D administration is still lacking. This leads however to a crucial unsettled point. A universally accepted definition of vitamin D deficiency/insufficiency (optimal blood levels) in IBD is still undefined, as the presently used normal range derives from studies centered on bone metabolism. The circulating levels required for disease prevention and management of IBD still need to be defined, as well as the optimal dosage for replacement and its duration. The vitamin D/VDR pathway represents however a promising area for further research, and a better understanding of its functions may lead to novel therapeutic strategies.

## Figures and Tables

**Table 1:** RCTs evaluating vitamin D supplementation on IBD and clinical course.

STUDY	Country	Patients Included	Disease	Intervention Group	Vitamin D Doses in the Intervention Group	Control Group	Control Group (Placebo or VitD)	Difference in the Mean Daily Dose *	Follow-Up	Outcomes (Disease Activity Evaluation)	Outcomes (Adverse Events; Vitamin D Levels)	Relapse Intervention vs. Control
Bafutto 2017 [101]	Brazil	30 moderate to severe and VitD levels < 30 ng/mL	CD	20	50,000 IU/die 10.000 IU/die	10	2000 UI/die	48,000 IU 8000 IU	8 weeks	↓CRP; ↓FC; ↑IBDQ	↑VitD	n.a.
Ahamed 2019 [102]	India	60 with UCDAI >3 and VitD levels < 40 ng/mL	UC	30	60,000 IU/d for 8 days	30	placebo	17,142 IU	4 weeks	↓UCDAI; ↓CRP; ↓FC	↑VitD; =AE	n.a.
Narula 2017 [103]	Canada	34 in remission	CD	18	10,000 IU/die	16	1000 IU/die	9000 IU	12 months	=CRP; ↓relapse **	↑VitD; =AE	0 vs. 3 **
Dadaei 2015 [104]	Ireland	108 and VitD levels < 30 ng/mL	IBD	53 (10 CD; 43UC)	50,000/week	55 (6 CD; 49UC)	placebo	7142 UI	12 weeks	none	↑VitD	n.a.
de Bruyn 2021 ** [105]	Netherlands and Belgium	143 with ileocolonic resection	CD	72	25,000/week	71	placebo	3571 UI	26 weeks	=Rutgerts score; =IBDQ; =CRP; =FC	↑VitD; =AE	n.a.
Sharifi 2016 [106]	Iran	86 in remission	UC	46	300,000 IU/90 die	40	placebo	3300 IU	3 months	↓ESR; ↓CRP	↑VitD	n.a.
Mathur 2017 [107]	U.S.A.	18 and VitD < 30 ng/mL	UC	10	4000 IU/die	8	2000 IU/die	2000 IU	3 months	=CRP; =pMayo; ↑SIBDQ	↑VitD; =AE	n.a.
Raftery 2015 [54]	Ireland	27 in remission	CD	13	2000 IU/die	14	placebo	2000 IU	3 months	=CDAI; =CRP; =FC; =QoL	↑VitD	0 vs. 0
Tan 2018 [108]	China	91 with VitD levels < 20 ng/mL	IBD	23 CD 24 UC	150,000 IU/90 die	19 CD 25 UC	placebo	1666 IU	12 months	=CRP; =ESR; =CDAI; =pMayo	↑VitD; =AE	n.a.
Bendix 2015 ‡ [109]	Denmark	18	CD	9 largest VitD increase ‡	1200 IU/die	9 seasonally matched ‡	placebo	1200 IU	26 weeks	=CRP; =HBI; =CDAI	↑VitD	0 vs. 1
Bendix-Struve 2010‡ [110]	Denmark	20	CD	10 largest VitD increase ‡	1200 IU/die	10 seasonally matched	placebo	1200 IU	12 months	=CDAI	↑VitD	0 vs. 1
Jorgensen 2010 [92]	Denmark	94 in remission	CD	46	1200 IU/d	48	placebo	1200 IU	12 months	↓relapse rate †	↑VitD; ↓AE	6 vs. 14
Bartels 2014 [111]	Denmark	19 in remission	CD	10 with increased in vitD levels	1200 IU/die	9 seasonally matched ‡	placebo	1200 IU	26 weeks	=CRP =CDAI	n.a.	0 vs. 1
Karimi 2019 [112]	Iran	46 II mild to moderate disease	UC	24	2000 IU/die	22	1000 IU/die	1000 IU	12 weeks	↓CDAI; ↓IBDQ	↑VitD; =AE	n.a.
Arihiro 2018 [113]	Japan	223	IBD	108	500/die	115	placebo	500 IU	2 months	↑UCDAI; ↓Lichtiger score;	↑VitD; =AE	n.a.

*: mean dose assumed in the intervention group, calculated as follows: (Intervention VitD dose − Control group VitD dose)/days in dose interval; AE: adverse events; CD: Crohn’s disease; UC: Ulcerative colitis; pMayo: partial Mayo score; SIBDQ: Short IBD questionnaire for quality of life; CDAI: Chron’s disease activity index; UCDAI: Ulcerative Colitis disease activity index; ESR: erythrocyte sedimentation rate; CRP: C-reactive protein; FC: Fecal Calprotectine; QoL: Quality of Life; HBI: Harvey–Bradshaw Index; n.a: not available. ** per-protocol analysis; † *p* = 0.06; ‡ drawn from the same population of Jorgensen 2010; ¶: Oral nano Vitamin D. Results showed in the study: ↑ = increase, ↓ = reduction, =no difference in the outcomes measured, respectively)

**Table 2:** Main limitations of the available studies.

Different populations –*Age* –*Ethnicity* –*Country* –*Sun exposure* –*Comorbidities*
Small number of patients
Partial data according to –*Disease subtype* –*Disease progression* –*Medical treatment* –*IBD-related surgery* –*Dietary pattern*
Limited data on changes in disease activity
Different vitamin D dosage regimens and treatment duration

## Data Availability

Not applicable.
